# Supplementary material for: Fluorescence-lifetime optical electrophysiology in contracting cardiomyocytes
Source: Proc Natl Acad Sci U S A. 2026 Jun 3;123(23):e2533881123. doi: 10.1073/pnas.2533881123 (PMC13250515; doi:10.1073/pnas.2533881123)
Supplement: Supplementary file 1 — Appendix 01 (PDF) [file pnas.2533881123.sapp.pdf]

Supporting Information for

Fluorescence-lifetime optical electrophysiology in contracting cardiomyocytes

E. Millar, E. Huethorst, V. Zickus, G. Astrauskaitė, J. Zhao, G. G. Taylor, C. Bruschini, E. Charbon, G. L. Smith, C. Müllenbroich, and D. Faccio

Caroline Müllenbroich, Daniele Faccio
E-mail: Daniele.Faccio@glasgow.ac.uk, Caroline.Muellenbroich@glasgow.ac.uk

This PDF file includes:

Supporting text
Figs. S1 to S10
Tables S1 to S3
SI References

Supporting Information Text

Data visualization

All fluorescence lifetime images shown (Fig. 3 in the main paper) are visualized by weighting (overlaying) the raw FLIM data with the corresponding intensity image (also captured by the SPAD). This is done by first mapping the lifetime values to RGB colors using a chosen colormap, producing a false-color image that represents the spatial distribution of fluorescence lifetimes. Separately, the associated intensity image is first processed by filling in dead pixels in the SPAD. These were identified by comparing the median-filtered versions of the image (with kernel sizes 3 and 11), and replaced by using the lower scale (3×3) median values to fill regions with abnormally high variation. Then, contrast limited adaptive histogram equalization (CLAHE) is applied. This enhances local contrast throughout the field of view (FOV) while avoiding overamplification of noise or saturation of bright regions. Finally, each RGB channel of the lifetime image is scaled by the enhanced intensity image, preserving the original color (lifetime) information but adjusting the brightness according to local intensity. This combined visualization improves the interpretability of spatial lifetime features compared to the raw FLIM images.

To compare fluorescence lifetime changes under experimental conditions, we first employ a classical normalization approach relative to a baseline frame, defined as

$$\Delta\tau/\tau_0 = \frac{\tau_n - \tau_0}{\tau_0}, \qquad [1]$$

where τ_n denotes the fluorescence lifetime in frame n, and τ_{baseline} is the mean pre-stimulus or reference lifetime. This global $\Delta\tau/\tau_0$ metric is widely used in electrophysiology studies to quantify changes over time and is effective in assessing action potential and calcium waveforms, as shown in Fig. S6*B*.

To isolate and visualize propagating features such as lifetime wavefronts, we additionally compute the normalized frame-to-frame gradient

$$\Delta\tau/\tau_{n-1} = \frac{\tau_n - \tau_{n-1}}{\tau_{n-1}}. \qquad [2]$$

This local temporal derivative emphasizes dynamic transitions between consecutive frames, enhancing the visibility of moving wavefronts, as shown in Fig. S6*A*, S5*A* and Fig. 5*M*, *N* of the main paper. This method is particularly useful for highlighting spatially confined transient changes that may not be apparent on the $\Delta\tau/\tau_0$ map.

Statistical analysis of pharmacological contraction suppression

In the following, we show the statistical analysis performed to quantify the reduction in contraction parameters after treatment of the cardiomyocyte monolayers with 20 mM 2,3-butanedione monoxime (BDM) corresponding to Fig. 2 of the main paper. These data were analyzed with the open source software MUSCLEMOTION (1). The box plots in Fig. S1*A* show a comparison between the four main physiological parameters before and after BDM treatment. The four parameters analyzed are contraction duration at 50% amplitude (CD_{50}), contraction duration at 90% amplitude (CD_{90}), rise time (T_{Rise}), and decay time (T_{Decay}). The corresponding values are annotated on Fig. S1*B*, *C* for the sample before and after BDM treatment, respectively. The two averaged waveform plots shown are normalized with respect to control (contractile) conditions. Due to the fact that both data sets were taken with the same illumination conditions, we can also compare the amplitudes of the normalized plots in Fig. S1*B*, *C* to determine that the BDM treatment reduced the contraction amplitude of the monolayer by 76.27%. For all analysis shown, temporal sample sizes of $n = 8$ beats were used for control and 5 beats for the BDM-treated group, and both data were averaged over a 204×204 µm FOV, completely populated with cells.

The t- and p-values for the four parameters analyzed are summarized in Table S1 .

Parameter	t-value	p-value
CD_{50}	28.66	1.09×10^{-11}
CD_{90}	8.82	2.55×10^{-6}
T_{Rise}	3.49	5.02×10^{-3}
T_{Decay}	5.62	1.54×10^{-4}

Table S1. t-values and p-values for all contraction parameters compared before and after BDM treatment.

Characterization and certificate of analysis of commercial hiPSC-CMs

The hiPSC-CMs used in this study were obtained from a commercial supplier and delivered as cryopreserved live cells. The differentiation protocol used by the supplier is proprietary and therefore cannot be disclosed in full detail. However, the certificate of analysis (COA) provided with the commercial cell batch includes key information regarding cell origin, reprogramming method, differentiation status, purity, electrophysiological properties, and quality control testing.

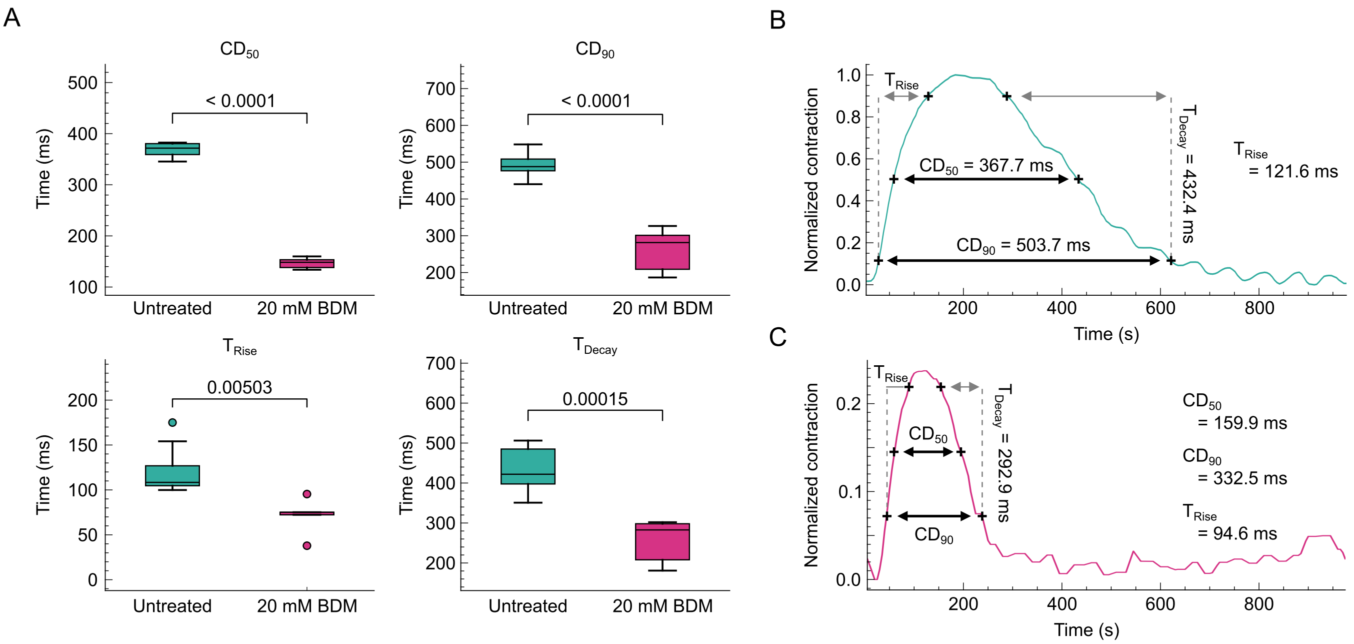

Fig. S1. Quantitative analysis of pharmacological contraction suppression. (*A*) Quantitative comparison of contractile parameters—contraction duration at 50% amplitude (CD_{50}), contraction duration at 90% amplitude (CD_{90}), rise time (T_{Rise}), and decay time (T_{Decay})—before and after BDM treatment. $n = 8$ for control and 5 for BDM-treated groups. The boxes show the IQR, the horizontal lines represent medians, and the whiskers extend to 1.5$\times$ the IQR. P-values are indicated directly on the plots and were obtained using two-sample independent t-tests. (*B*) Average contraction waveform of an untreated sample, annotated with the parameters analyzed in (*A*). (*C*) Average contraction waveform of a sample after motion suppression with 20 mM BDM, annotated with the parameters analyzed in (*A*).

Cells were derived from human skin fibroblasts obtained from a Caucasian male donor and reprogrammed using a Sendai virus-based method. This approach is non-integrating and certified for viral clearance. The resulting induced pluripotent stem cells were differentiated into cardiomyocytes using the supplier's proprietary differentiation protocol.

Cells were supplied frozen in cryopreservation medium and stored at $-150°C$ in liquid nitrogen vapor or liquid phase until use. Quality control analyses performed by the supplier include cardiomyocyte identity verification (cTnT+ staining), purity assessment, electrophysiological characterization, pharmacological response testing, and sterility screening.

Electrophysiological measurements were obtained using field potential recordings. Drug response assays included exposure to the potassium channel blocker E4031, the calcium channel blocker nifedipine, and the sodium channel blocker mexiletine, confirming expected pharmacological responses. Sterility testing confirmed the absence of mycoplasma and other detectable contaminants.

A summary of the COA specifications provided by the manufacturer for the batch used in this study is presented in Table S2.

Parameter	Specification
Product format	Live cells frozen in cryopreservation medium
Source	Differentiated human iPSC
Storage	$-150°C$ (gas/liquid phase LN_2)
Origin cell line	Human skin fibroblast, Caucasian male donor
Reprogramming method	Sendai virus-based reprogramming (non-integrating, virus clearance certified)
Differentiation protocol	Proprietary protocol
Identity	Cardiomyocytes (cTnT+ cells)
Purity	99.3%
Live cell count	7.54×10^6 cells
Viability	71.4%
Beat rate	33.29 bpm
Field potential duration (FPD)	322.2 ms
Corrected FPD (FPDcF)	265.4 ms
Drug response: E4031	FPDcF increased $>$15% at 0.01 µM (Pass)
Drug response: Nifedipine	FPDcF increased $>$30% at 0.01 µM (Pass)
Drug response: Mexiletine	Spike amplitude decreased $>$15% at 10 µM (Pass)
Mycoplasma test	Negative
Sterility (other contaminants)	Negative

Table S2. Summary of certificate of analysis specifications provided by the manufacturer for the commercial hiPSC-CMs used in this study.

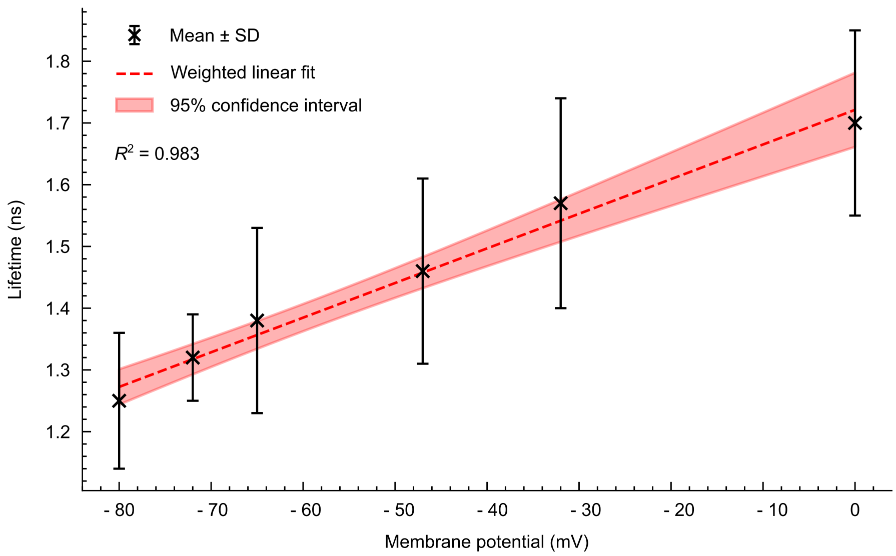

Fig. S2. Multipoint calibration of fluorescence lifetime versus membrane potential in H9C2 cells. Fluorescence lifetimes of FluoVolt–loaded H9C2 cells measured at discrete valinomycin-induced membrane potentials ranging from -80 to 0 mV. Scattered points represent the mean lifetime over ~ 10 cells per condition, with error bars indicating the standard deviation (SD). A weighted linear regression (red dashed line) defines the calibration relationship between fluorescence lifetime and membrane potential, with the 95% confidence interval shown as a shaded region. The fit demonstrates good linearity ($R^2 = 0.983$), enabling interpolation of membrane potential from lifetime measurements.

Calibration of fluorescence lifetime to membrane potential

In this section, we provide additional details on the calibration procedure used to translate fluorescence lifetime values into membrane potential ($\mathrm{V_m}$) readings, following protocols presented in (2). Figure S2 shows a scatter plot in which each point represents the mean fluorescence lifetime measured in H9C2 cells at a given $\mathrm{V_m}$ (cells were in a confluent monolayer so the numbers varied between imaging regions but $n \approx 10$ cells were analyzed per polarization condition). The relationship between $\mathrm{V_m}$ and the fluorescence lifetime was fitted with a weighted linear regression which serves as the reference framework to interpolate $\mathrm{V_m}$ in hiPSC-CM monolayers.

The errors in $\mathrm{V_m}$ derived from the calibration of the fluorescence lifetime are comparable with previously reported values using similar lifetime-based estimation (3), with a root mean square error ranging from 14 mV to 31 mV. While the overall error magnitude is comparable to previous reports, the slightly higher values observed here likely reflect a combination of factors including the high frame rates used, the simplified lifetime estimation approach (rather than full time-correlated single-photon counting fitting), and the use of valinomycin-mediated $\mathrm{V_m}$ manipulation in place of voltage clamping.

Uncertainties in $\mathrm{V_m}$ were estimated by propagating errors through the inverse linear calibration using confidence intervals derived from the weighted linear regression. Contributions from the measured fluorescence lifetime variability between cells at each $\mathrm{K^+}$ level, as well as uncertainties in the fitted calibration, were incorporated into these intervals, which were then mapped through the inverse calibration using interpolation (with extrapolation beyond the calibrated range where necessary) to yield a per-measurement uncertainty in $\mathrm{V_m}$. These uncertainties are visualized as shaded confidence bands in Fig. 3 of the main text.

Inspection of residuals revealed no systematic deviation from linearity, with residuals randomly distributed around zero and no evidence of structured trends across the calibration range. Additionally, the goodness of fit (R^2) was 0.983, confirming that the linear model adequately describes the calibration data.

Quantitative analysis of action potential waveforms

To quantify AP variations, we compared the averaged AP waveforms for multiple ROIs within two hiPSC-CM monolayers from the same plating. The table in Fig. S3 reports the key parameters for the time-averaged waveforms from the traces shown in Fig.3 of the main paper, where early, middle and late repolarization are defined as the duration from APD $10-30$, $40-60$, and $70-90$, respectively. These parameters are annotated on an example time-averaged trace in Fig. S3. As can be seen, the values for any given single AP waveform can differ within the same sample. The data reported in the table were calculated from interpolated time traces to remove discretization from the camera frame rate.

Sub-FOV action potential waveforms

To examine the variability of the observed late-phase repolarization heterogeneity, we extracted AP waveforms within individual ROIs across the FOV shown in Fig. 3H in the main paper. Figure S4A shows the representative FLIM image averaged across all frames with four ROIs highlighted. For each ROI, we extracted individual AP traces, temporally aligned them to the peak depolarization, and computed the normalized, temporally averaged waveform (Fig. S4B–E). Each trace shows qualitatively

	Monolayer 1 (ms)		Monolayer 2 (ms)	
	ROI 1	ROI 2	ROI 1	ROI 2
APD_{90}	337.14	254.01	362.09	213.22
T_{Rise}	17.09	18.48	14.75	14.80
Early rep.	111.68	85.51	89.69	58.79
Mid rep.	33.11	26.11	24.50	17.84
Late rep.	29.48	25.08	71.48	25.07

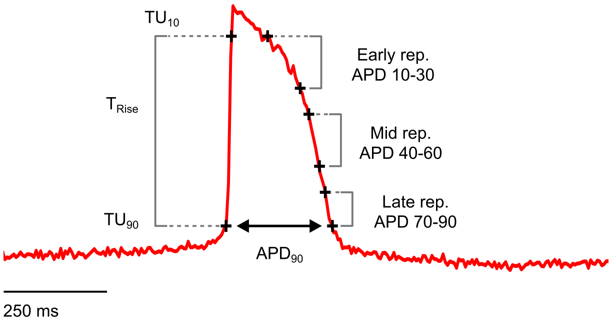

Fig. S3. Comparison of action potential parameters across multiple regions of two monolayers from a single plating. Table of action potential waveform values quantitatively analyzed for the data shown in Fig. 3 of the main paper alongside a representative time-averaged waveform (Fig. 3*G* in the main paper) annotated with the parameters analyzed.

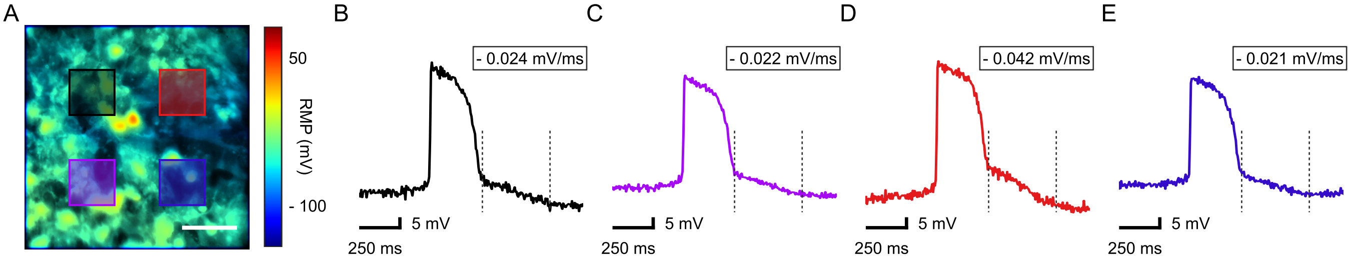

Fig. S4. Examples of AP waveforms across a single FOV to highlight non-uniform late-phase repolarization dynamics. (*A*) Representative FLIM image with four ROIs highlighted. White scale bar, 50 µm. (*B*–*E*) Normalized and temporally averaged AP traces from each ROI. Each trace represents the mean waveform across multiple APs within the ROI, highlighting distinct late-phase repolarization dynamics across regions. Annotated values denote the dV/dt within the regions bounded by the dashed lines after phase 3 repolarization.

different late-phase behavior, underscoring the spatial heterogeneity across the monolayer. To quantify this variation, we calculated the dV/dt for the late-phase stage for each trace. The results of this analysis are annotated in Fig. S4*B*–*E*.

Imaging calcium wave propagation with fluorescence lifetime

In Table S3 we show the quantitative analysis of a representative calcium transient from midway through the recording in Fig. 5F in the main paper.

Parameter	Value (ms)
CaT_{90}	494.79
CaT_{50}	296.88
T_{Rise}	83.33
T_{Decay}	286.46

Table S3. Calcium transient parameters measured in a representative beat located halfway through the recording in Fig. 5*F* of the main paper.

Furthermore, to directly compare the fluorescence lifetime analysis with conventional intensity-based readouts, we examined the propagation of calcium waves in the cardiomyocyte monolayers using the fluorescence lifetime signal. Although the main text (Fig. 5) shows the frame-by-frame dynamics of $\Delta F/F$ and the corresponding activation map extracted from the intensity data, here we present an analogous analysis performed on the lifetime signal. Figure S5a shows the evolution of $\Delta\tau/\tau_{n-1}$ across the field of view during sequential beats. The activation maps derived from the fluorescence lifetime and from intensity (Fig. S5*B*) demonstrate that both modalities capture the same spatiotemporal propagation patterns of calcium activation throughout the tissue. As expected, the signal-to-noise ratio of the lifetime traces is lower than that of intensity, but the essential features of wave propagation remain accessible, confirming that the FLIM signal can be used for quantitative mapping of calcium activation dynamics.

Widefield imaging of action potential propagation

To showcase our system's ability to spatially resolve action potential (AP) wave propagation, we imaged a hiPSC-CM monolayer stained with FluoVolt across an 816 × 816 µm FOV. Figure S6*A* illustrates the spatial mapping of AP propagation captured with our system with the corresponding AP time trace shown in Fig. S6*B*. In this experiment, we applied our temporal cleaning

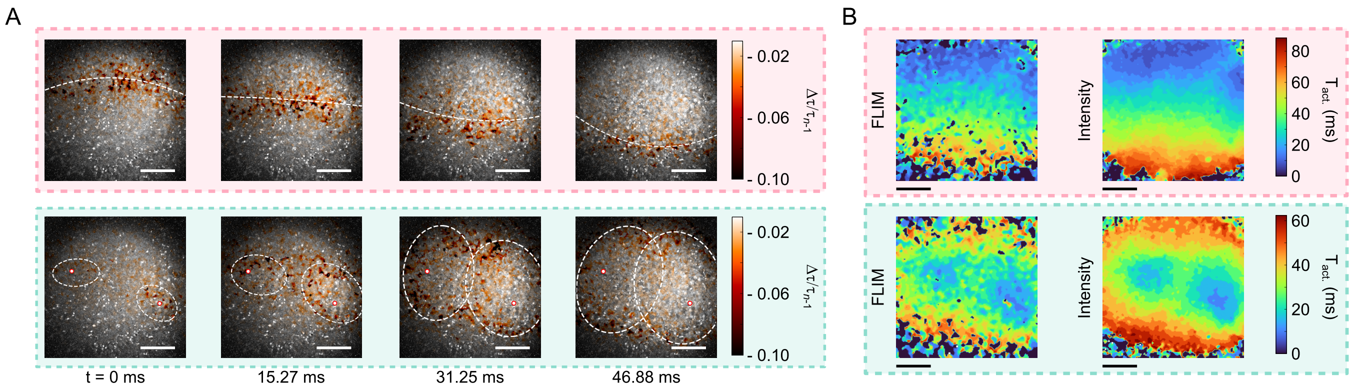

Fig. S5. Calcium wave propagation analyzed from fluorescence lifetime signals. (*A*) Snapshots of $\Delta\tau/\tau_{n-1}$ frames corresponding to the time trace in Fig. 5 of the main paper. Colored boxes (pink, green) highlight the same beats marked in Fig. 5. (*B*) Activation maps showing the time of calcium activation ($T_{act.}$) extracted from fluorescence lifetime (left) and intensity (right). Both approaches reveal consistent propagation patterns, though with reduced signal-to-noise in the lifetime data.

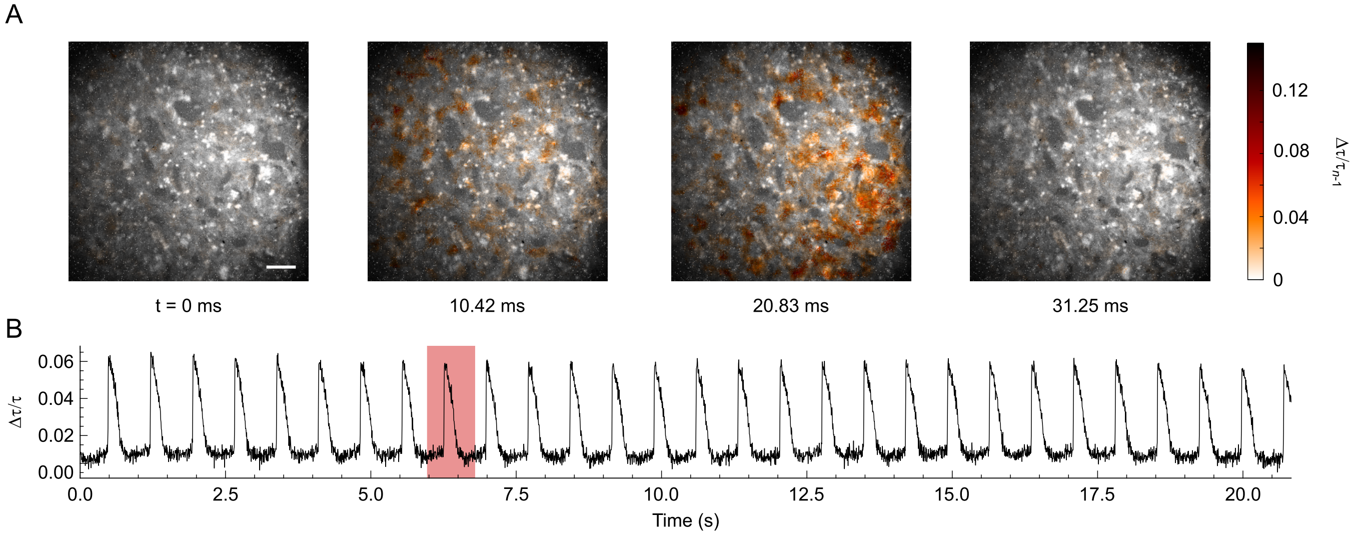

Fig. S6. Widefield fluorescence lifetime imaging of action potential propagation in a hiPSC-CM monolayer. (*A*) $\Delta\tau/\tau_{n-1}$ time-lapse frames showing the propagation of the action potential wavefront in the hiPSC-CM monolayer. White scale bar, 100 µm. (*B*) Action potential time trace recorded over 20 s with the beat shown in (*A*) highlighted in red.

process to enhance the clarity of the recorded signals. The refined recordings allow us to visualize the sequence of activation with high fidelity.

Figure S6*A* shows a series of frames that map the progression of the action potential wavefront ($\Delta\tau/\tau_{n-1}$). Each frame has been processed to minimize temporal noise, as stated above. These detailed visualizations confirm the ability of our system to capture rapid electrophysiological events.

The reduced amplitude of the lifetime transient in Fig. S6(*B*), relative to the data shown in the main text, arises from lower photon counts in the demagnified configuration, which affects the accuracy of absolute lifetime estimation but does not impact the spatiotemporal mapping shown in Fig. S6(*A*).

Calibration of fluorescence lifetime to calcium ion concentration

Here we show the calibration curve used to convert fluorescence lifetime data into quantitative intracellular Ca^{2+} concentrations. A series of calibration solutions were generated by mixing Ca-EGTA and EGTA buffers with 1 µM Cal-520, potassium salt, yielding free Ca^{2+} levels ranging from 0.2 nM to 25 µM. A 200 µL aliquot of each solution was dispensed into a 96-well glass bottom plate and imaged using our FLIM system and a commercially available CellOPTIQ system (Clyde Biosciences, considered a gold standard for cardiac electrophysiology).

First, to ensure that the measurements from our system agree with standard calibration practices, we measured the fluorescence intensity of each well on a CellOPTIQ system to obtain the standard sigmoidal calibration curve reported for Cal-520, AM (4). We then repeated this measurement on our system using the time-integrated intensity images from the SPAD as a comparison. These two data are shown in Fig. S7*A* with the sigmoidal fit generated for the CellOPTIQ data. This fit gives a dissociation constant (K_d) of 186 ± 3 nM. The strong agreement between these data points confirms that the samples

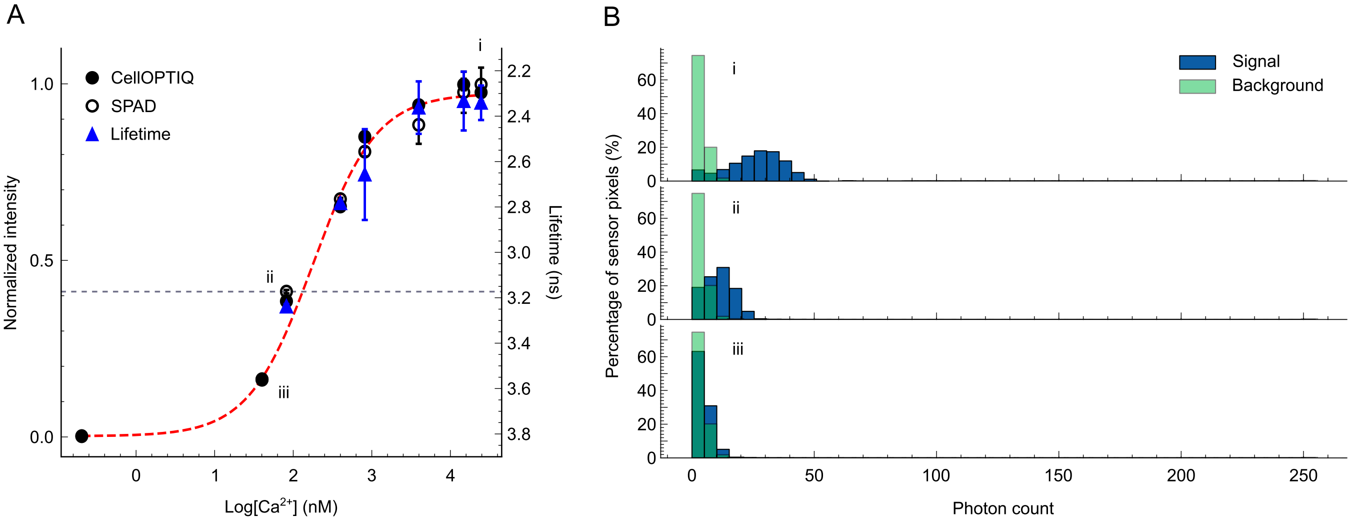

Fig. S7. Calibration curves relating fluorescence emission to calcium ion concentration and SPAD noise analysis. (*A*) Normalized fluorescence intensity of Cal-520, AM measured with both CellOPTIQ system (solid circles) and the SPAD array (open circles) at varying free Ca^{2+} concentrations. The sigmoidal curve is fitted to the CellOPTIQ data but shows good agreement with both imaging systems. Fluorescence lifetime values (blue triangles) are also plotted down to a threshold (gray dashed line) below which noise dominates and FLIM measurements are no longer reliable. Error bars represent the standard deviation. (*B*) Histograms of photon counts captured by the SPAD for the three points highlighted in (*A*). Below log[Ca^{2+}] values of 1.9 nM (iii) the distribution of the noise (green) becomes indistinguishable from that of the signal (blue). This overlap limits the ability to accurately extract calcium-dependent fluorescence lifetime changes at very low ion concentrations, imposing a lower threshold on the measurements in (*A*).

behaved similarly in both imaging systems for the full duration of the experiment. We then evaluated the average fluorescence lifetime of each well using the two gated images output by the SPAD, captured synchronously with the SPAD intensity data. In other words, the lifetime values blue triangles in Fig. S7*A* correspond directly to the well-documented intensity behavior of the dye (black circles).

Figure S7*B* shows histograms of the intensity data (photon counts) measured by the SPAD at three different free calcium concentrations – $\log[\mathrm{Ca}^{2+}] = 4.4$, 1.9 and 1.6 nM. At the lowest concentration (1.6 nM), the distribution of signal photon counts shows a complete overlap with the distribution of the background noise, making it impossible to distinguish noisy pixels from those containing a true signal. This sets a practical lower threshold for FLIM-based measurements, indicated in Fig. S7*A* with a gray dashed line. Any estimate of fluorescence lifetimes below this threshold would be inaccurate and dominated by noise. Importantly, our system still provides accurate lifetime values in physiologically relevant calcium ranges.

To ensure consistency between FLIM and intensity-based calibrations, we also used the sigmoidal fit derived from the CellOPTIQ intensity data to estimate the fluorescence lifetime behavior across all calcium concentrations. Using the dissociation constant ($K_d = 186$ nM), the mean measured lifetime at maximal calcium ($\tau_{\mathrm{max.}} = 2.41$ ns) and the interpolated lifetime at K_d ($\tau = 3.10$ ns), we calculated the lifetime of the calcium-free form of the dye as $\tau_{\mathrm{min.}} = 3.76$ ns. These values define a sigmoidal relationship between fluorescence lifetime and calcium concentration, modeled by the equation

$$[\mathrm{Ca}^{2+}] = K_d \cdot \frac{\tau - \tau_{\mathrm{min.}}}{\tau_{\mathrm{max.}} - \tau}. \qquad [3]$$

This overlapping calibration curve confirms the strong correspondence between intensity- and lifetime-based measurements, providing a robust method for converting FLIM data to intracellular calcium concentrations. These results validate the performance of our FLIM system for calcium quantification and confirm that Cal-520, AM exhibits consistent, well-characterized behavior across both imaging platforms when measured in solution. For all intracellular measurements presented in the main manuscript, we calibrated our time-course data using the established intracellular K_d for Cal-520, AM (320 nM) (4), which accounts for the altered binding affinity of the dye in intracellular environments.

To quantify the uncertainty in the inferred Ca^{2+} concentrations, we propagated the experimental uncertainty in the fluorescence lifetime measurements obtained during calibration through this mapping. Specifically, the standard deviation of the measured lifetimes at each calibration point (Fig. S7*A*) was used to estimate a representative lifetime uncertainty, which was then propagated analytically via the derivative of the calibration function with respect to lifetime. This yields an uncertainty in Ca^{2+} concentration that reflects the sensitivity of the nonlinear mapping to measurement noise in the lifetime estimates.

Rapid lifetime determination gating

In Fig. S8 we show examples of the gate and antigate profiles of the SwissSPAD3 camera. Figure S8*A*, *B* show examples of the fixed gate positions used with simulated fluorescence decays to demonstrate the data capture paradigm. Each gate counts the number of photons arriving while it remains open, illustrated by the highlighted regions under the blue decay curves.

The accuracy of the RLD approach depends on the precise timing of the gate relative to the fluorescence decay, with the highest accuracy coming from gating up to approximately the expected lifetime. To enable rapid lifetime estimation, a lookup

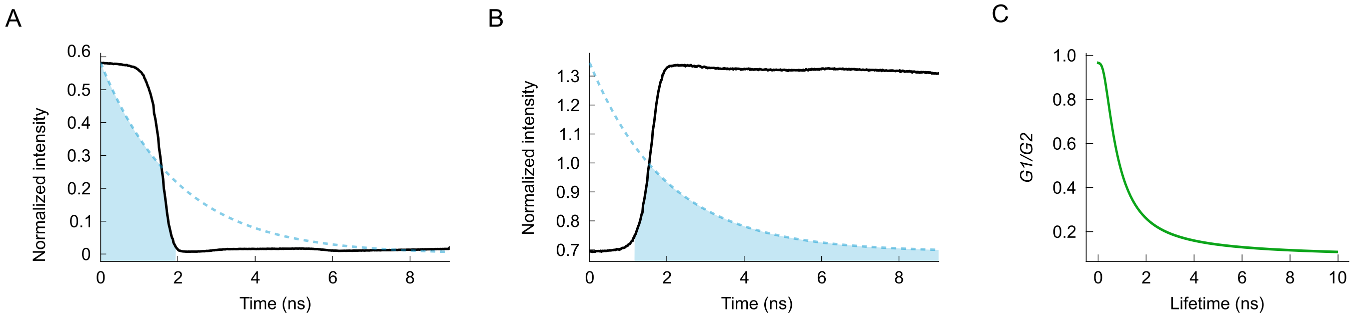

Fig. S8. Gate profiles and lookup table for rapid lifetime determination. (*A*, *B*) Gate (*A*) and antigate (*B*) profiles from the SPAD overlaid with simulated fluorescence decay for visualization of data capture. The shaded regions represent the different phases of the fluorescence decay captured by each channel. (*C*) Example lookup table relating the ratio between gate and antigate channels with fluorescence lifetime.

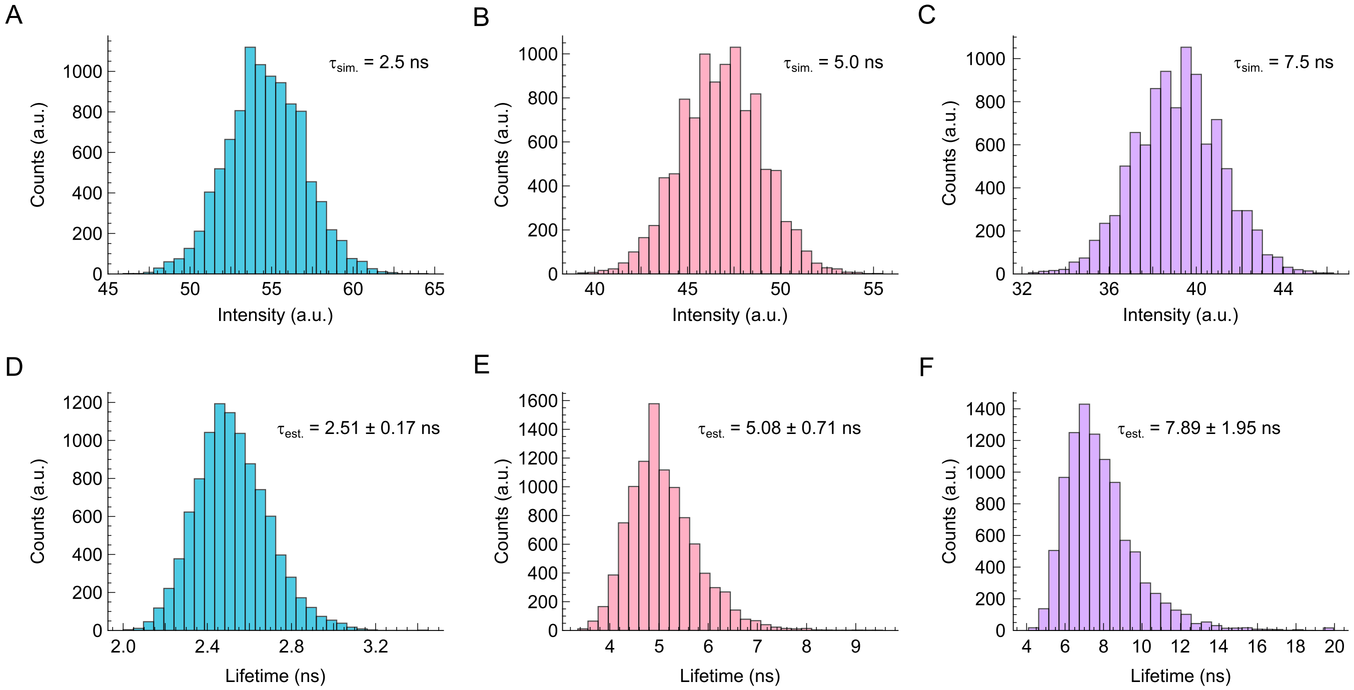

Fig. S9. Impact of Poissonian noise on dual-gated lifetime estimates. Histogram of total photon counts (gate + antigate) over 1,000 simulations for (*A*) τ =2.5 ns, (*B*) $\tau = 5.0$ ns and (*C*) τ =7.5 ns. (*D—F*) Corresponding histograms of lifetime estimates from the dual-gated RLD method: (*D*) $\tau = 2.5$ ns, (*E*) $\tau = 5.0$ ns, (*F*) $\tau = 7.5$ ns. The increasing spread and asymmetric tail at longer τ reflect how this method begins to fail when the detected lifetime deviates greatly from the fixed gate width ($\sim$ 2 ns here).

table is used to map the measured gate-to-antigate ratio ($G1/G2$) directly to the fluorescence lifetime, as illustrated in Fig. S8*C*. This calibration captures the expected ratio-lifetime relationship for the example gate configuration shown in Fig. S8*A*, *B*. The calibration of the lookup table is most effective when the ratio-lifetime curve has a steep slope in the expected lifetime range, so that even small changes in the measured ratio correspond to clear changes in lifetime. This slope and its position are optimized by approximately matching the gate duration to the anticipated lifetime.

Poissonian noise statistics in intensity and lifetime measurements

To illustrate how Poissonian noise in photon counts propagates into the distribution of lifetimes estimated by the RLD method, we performed 1,000 Monte Carlo simulations of monoexponential decay curves at three nominal lifetimes ($\tau_{\mathrm{sim.}} = 2.5$, 5.0 and 7.5 ns). Photon arrival times were binned into two gates (experimentally measured for our SPAD and averaged across the sensor area) and sampled according to a Poisson process with a fixed noise level. Histograms of the summed counts across both gates (Fig. S9*A*–*C*) demonstrate the intrinsic shot-noise-limited variance of the Poisson distribution under identical conditions.

When these noisy counts are converted to lifetime estimates using the dual-gated RLD method outlined above, the resulting lifetime distributions (Fig. S9*D*–*F*) show the same Poissonian statistics. For $\tau = 2.5$ ns (*D*), the estimate distribution remains approximately Gaussian, while for $\tau = 5.0$ ns (*E*) the spread increases, and for $\tau = 7.5$ ns (*F*) the deviation of the lifetime from the fixed gate interval further amplifies variability (manifested as an asymmetric tail). This transfer of count variation into lifetime uncertainty explains the increased variation in τ observed in our calcium calibration measurements (Fig. S7) under shot-noise limited conditions.

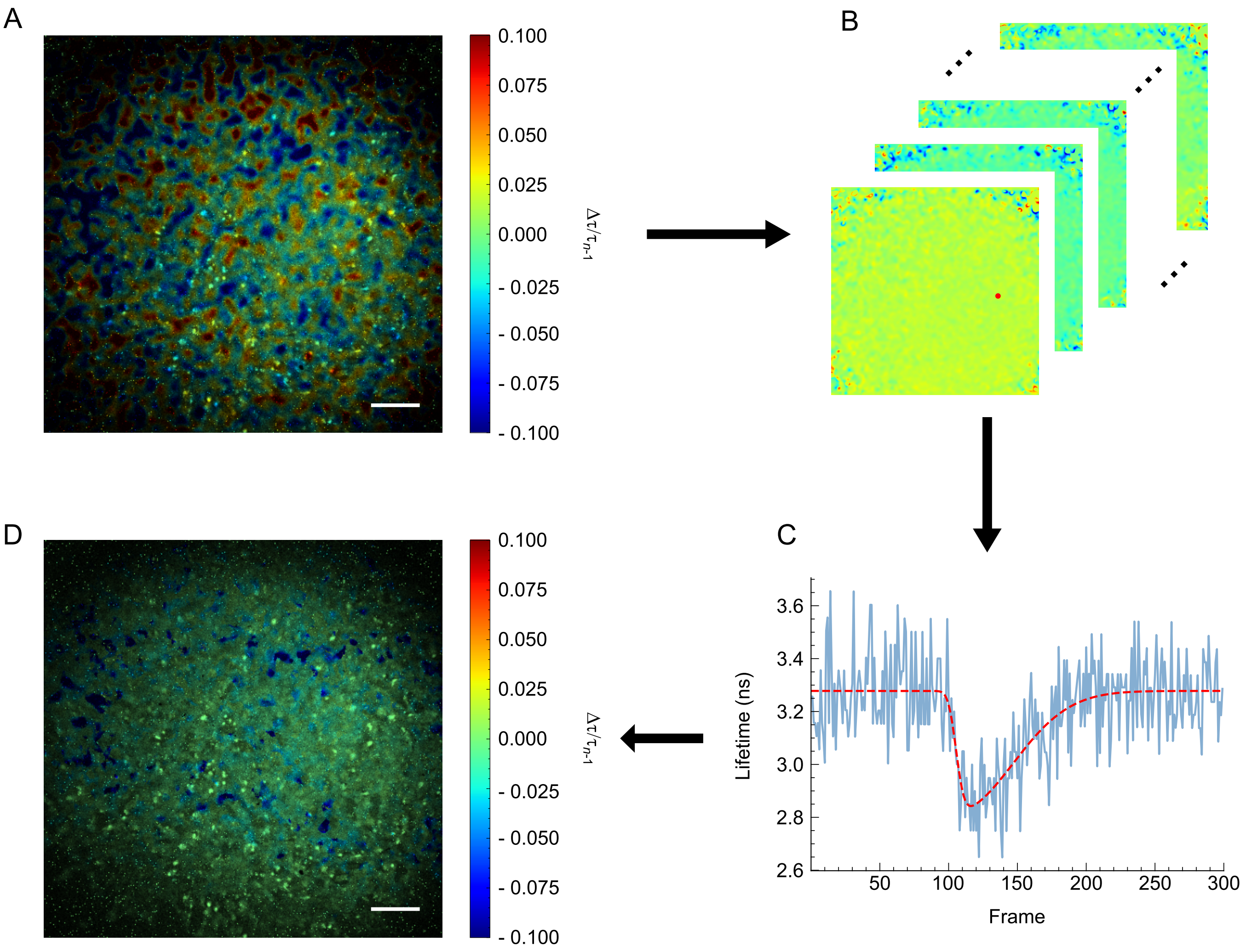

Fig. S10. Temporal cleaning of three-dimensional data cube. (*A*) $\Delta\tau/\tau_{n-1}$ image from uncleaned FLIM data. (*B*) Raw FLIM frames. (*C*) Single pixel (red in (*B*)) showing calcium wave and fitted curve. (*D*) Spatially cleaned frame showing $\Delta\tau/\tau_{n-1}$ wavefront (blue) with no noise. White scale bar, 200 μm.

The lookup table is generated to be optimized for the experimental data with a gate width of ~ 2 ns, such that using it for longer lifetimes as seen here would be impractical. This is illustrated by the increasing standard deviation on $\tau_{\text{est.}}$ shown in Fig. S9*D*–*F*. If one wished to examine longer lifetime samples experimentally, a different gate width should be chosen and a new lookup table generated.

Temporal cleaning

Here, we show pictorially the steps of the temporal cleaning procedure, used to suppress fluctuations in three-dimensional spatially resolved data cubes (originally reported in (5)). Due to frame-to-frame variations in the number of photons collected, the pixel-wise lifetime calculated will fluctuate from frame-to-frame over the course of a video. When plotting the instantaneous change in wavefront position ($\Delta\tau/\tau_{n-1}$) or traditional comparisons to a baseline frame ($\Delta\tau/\tau_0$), these fluctuations will present more obviously and obscure the desired signal with noise, as seen in Fig. S10*A*. By isolating a single pixel (Fig. S10*B*) and plotting its time-trace during physiological activity (Fig. S10*C*) we see the effect of these rapid fluctuations. By fitting an analytical expression to this waveform, we can effectively remove the noise and completely suppress variations from pixels where electrical activity is not observed. To approximate both calcium and action potential waves, we fit a skew-normal distribution of the form

$$f(x) = 2\phi(x)\,\Phi(\alpha x)\,, \qquad [4]$$

where

$$\phi(x) = \frac{1}{\sqrt{2\pi}} e^{-\frac{x^2}{2}}\,, \qquad [5a]$$

$$\Phi(x) = \int_{-\infty}^{x} \phi(t)\,\mathrm{d}t = \frac{1}{2}\left[1 + \mathrm{erf}\left(\frac{x}{\sqrt{2}}\right)\right]. \quad [5b]$$

The shape of the distribution is controlled by α, with $\phi(x)$ and $\Phi(x)$ representing the standard normal probability density and the cumulative distribution functions, respectively. This fitted data cube can then be visualized (Fig. S10*D*) to show spatially resolved fluorescence lifetime changes devoid of noise fluctuations.

References

1. Sala L, et al. (2018) MUSCLEMOTION. *Circulation Research* 122(3):e5–e16.
2. Cerignoli F, et al. (2012) High throughput measurement of Ca^{2+} dynamics for drug risk assessment in human stem cell-derived cardiomyocytes by kinetic image cytometry. *Journal of Pharmacological and Toxicological Methods* 66(3):246–256.
3. Lazzari-Dean JR, Gest AM, Miller EW (2019) Optical estimation of absolute membrane potential using fluorescence lifetime imaging. *eLife* 8.
4. AAT Bioquest, Inc. (2025) Cal-520®, AM Product Datasheet (https://www.aatbio.com/products/cal-520-am). Accessed: 2025-04-24.
5. Gariepy G, et al. (2015) Single-photon sensitive light-in-fight imaging. *Nature Communications 2015 6:1* 6(1):1–7.
